# Transcranial sonography for diagnosis of Parkinson's disease

**DOI:** 10.1186/1471-2377-10-9

**Published:** 2010-01-21

**Authors:** Sabine Mehnert, Iris Reuter, Karsten Schepp, Peter Maaser, Erwin Stolz, Manfred Kaps

**Affiliations:** 1 Department of Neurology, Justus-Liebig University of Giessen, Giessen, Germany

## Abstract

**Background:**

In idiopathic Parkinson's disease (IPD) transcranial sonography (TCS) represents an alternative diagnostic method to verify clinical diagnosis. Although the phenomenon of an increased echogenicity of the Substantia nigra (SN) is well known this method is still not widly used in the diagnostic workup. Until now reliability of this method is still a matter of debate, partly because data only existed from a few laboratories using the same ultrasound machine. Therefore our study was conducted to test the reliability of this method by using a different ultrasound device and examining a large population of control and IPD subjects by two examiners to calculate interobserver reliability.

**Method:**

In this study echogenicity of SN was examined in 199 IPD patients and 201 control subjects. All individuals underwent a neurological assessment including Perdue pegboard test and Webster gait test. Using a Sonos 5500 ultrasound device area of SN was measured, echogenicity of raphe, red nuclei, thalamus, caudate and lenticular nuclei, width of third and lateral ventricle were documented.

**Results:**

We found a highly characteristic enlargement of the SN echogenic signal in IPD. The cut-off value for the SN area was established using a ROC curve with a sensitivity of 95% corresponding to an area of SN of 0.2 cm^2 ^and was found to be equivalent to the cut-off values of other studies using different ultrasound devices.

**Conclusions:**

Our study shows that TCS is a reliable and highly sensitive tool for differentiation of IPD patients from individuals without CNS disorders.

## Background

Diagnosis of idiopathic Parkinson's disease (IPD) is usually based on clinical criteria. However, in some cases differential diagnosis especially in early stages of the disease might be difficult. Single photon emission tomography (SPECT) or positron emission tomography (PET) may pose problems in terms of costs, availability and exposure to radiation. In recent years, transcranial B-mode sonography (TCS) appeared as new diagnostic modality to support the clinical diagnosis of IPD using a different physical principle of imaging. Since the first and pathbreaking description of an increased echogenicity of the substancia nigra (SN) in IPD by Becker et al in 1995, these findings were corroborated by several studies since then [1-9]. However, TCS is not a widespread diagnostic method, because patient and control cohorts so far were small and diagnostic thresholds appeared to be dependent on the technology used. In fact, reference values in large patient groups so far have been only established for Siemens Sonoline Elegra (Siemens, Erlangen, Germany) and lately for Aplio (Toshiba Medicals, Japan) [2,3,8,10]. We used a different ultrasound device, SONOS 5500, Philips, Netherlands, but followed an established examination protocol. Until now studies using the same ultrasound machine only involved small subject groups. Vlaar et al examined 82 patients with unclassified Parkinsonian syndromes using a SONOS 5500 and reported a sensitivity of 50% to differentiate IPD patients from patients without nigrostriatal degeneration or atypical Parkinsonian syndromes (specificity 82% and 43% respectively). Remarkably a positive qualitative scoring of the SN echogenicity always corresponded to a SN area larger than 0.2 cm^2 ^[11]. Hagenah et al reported a sensitivity of 0.71 and a specificity of 0.58 at a cut-off of 0.27 cm^2 ^SN area examining 58 individuals, 21 controls, 24 with clinically definite PD and 13 unaffected Parkin gene carriers [12]. In another study an increased area of SN in a group of 20 family members with a PINK1 mutation and 15 relatives of patients with sporadic PD compared to 18 healthy subjects was documented [13]. The current study examines the echogenicity of the SN in IPD in a very large cohort of patients and controls. Aim of this study was to clarify the influence of age or life circumstances on the echogenicity of SN in healthy and IPD individuals. A further aim was to establish reference values beyond the most often used ultrasound technology and to provide data on the interobserver reliability of the method. This ultrasound technique has often been criticized as subjective, as observer and device dependent.

## Methods

**Figure 1:**
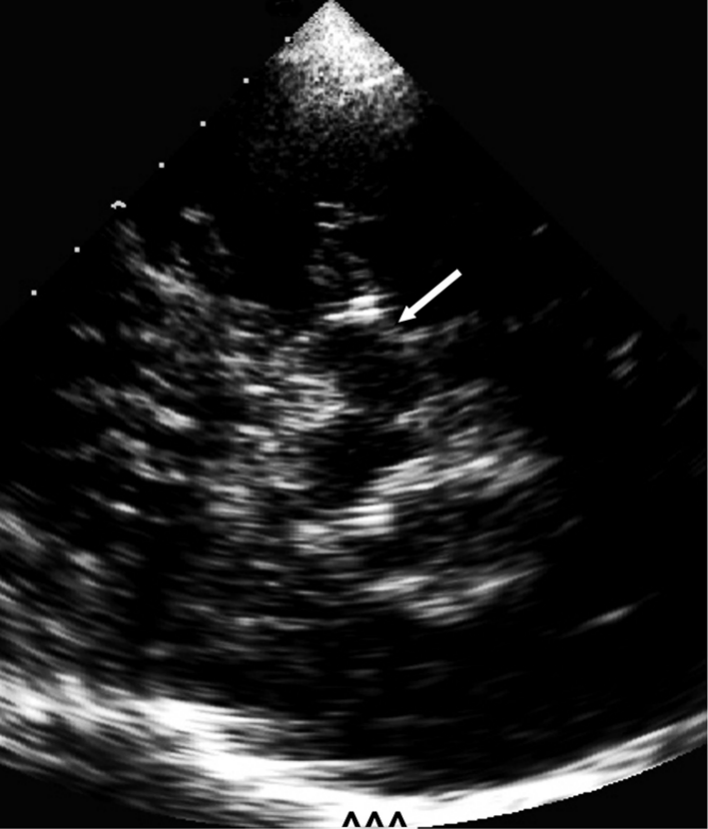
**TCS image of mesencephalon of healthy individual. **In the center the hypoechogenic mesencephalon (arrow) with small hyperechogenic SN is shown. Contralateral skull (^^^).

**Figure 2:**
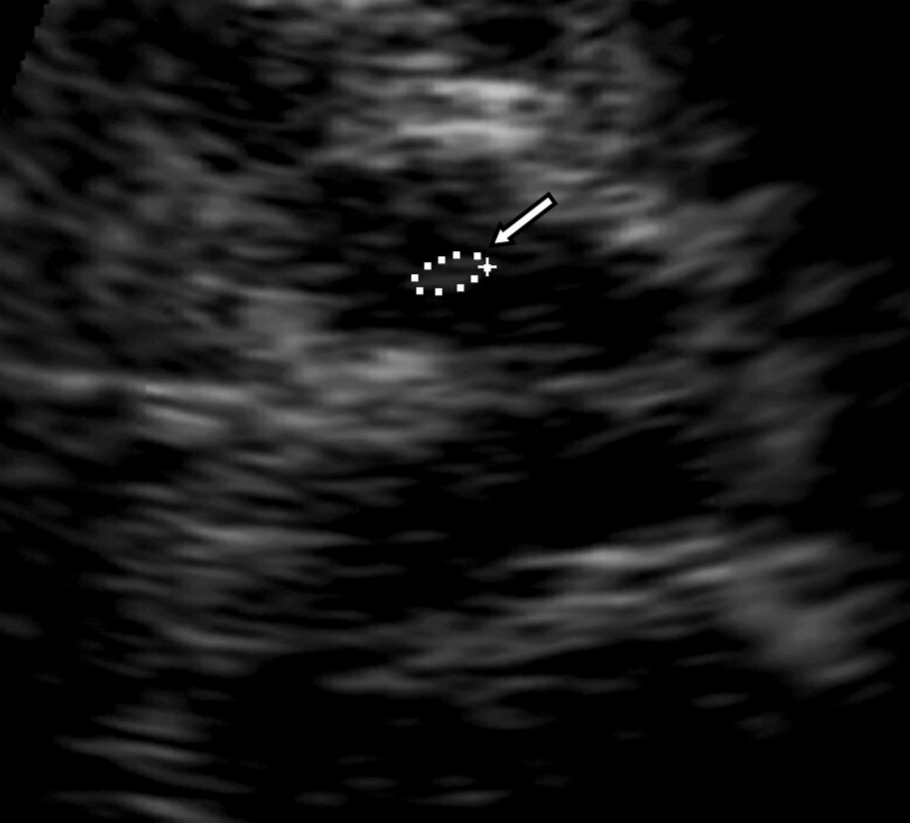
**TCS image (zoom) of butterfly shaped mesencephalic brainstem of healthy individual, left SN (arrow) with an area of 0.10 cm^2^**.

**Figure 3:**
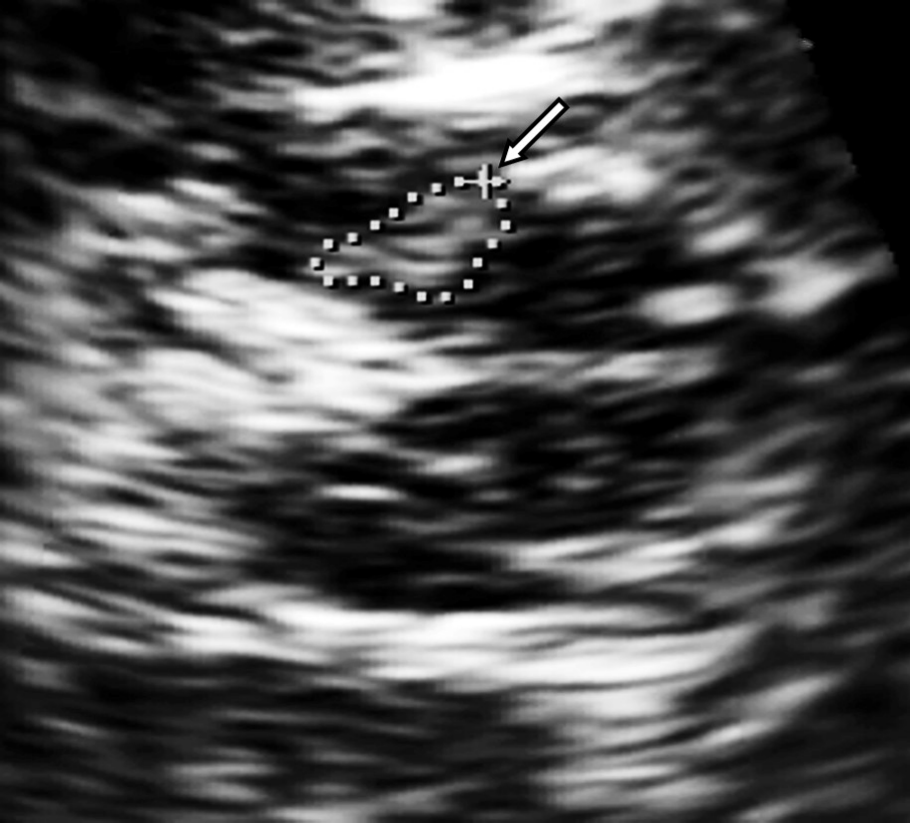
**TCS image (zoom) of mesencephalic brainstem of patient with IPD, left SN (arrow) with an area of 0.43 cm^2^**.

The group of IPD patients consisted of 199 individuals (131 males and 68 females). Diagnosis was established according to the United Kingdom brain bank criteria [14]. Participants were consecutive patients recruited from the Parkinson clinic Bad Nauheim and from the University hospital Giessen. The control group consisted of 201 individuals (89 males, 112 females) with no known central nervous disease (NCD). 140 of them were either university students or clinic staff members, 61 were patients with peripheral nerve disorders or musculosceletal diseases or visitors. Controls were chosen, so that at least 30 individuals per decade from age 20 to 79 could be examined. All participants gave written informed consent according to the Declaration of Helsinki. The study was approved by the ethics committee of the University clinic Giessen, Germany. All individuals were seen by an experienced neurologist (SM, IR) and underwent a thorough neurologic examination including Unified Parkinson's Disease Rating Scale (UPDRS) part I-IV, Webster's gait test [15,16]. Purdue's pegboard test was performed in all subjects to quantify hand motor function [17]. The medical and social history was taken from all individuals including date and area of birth, exposure to toxicologic substances (wood preservatives, solvents, pesticides), nutrition (regular continental, vegeterian, protein-rich), diseases, medication, use of alcohol or nicotin. For IPD patients onset and duration of disease, start, preparation and dose of medication as well as side effects such as dyskinesia were documented. A Levodopa Equivalent Dose (LED) was estimated [18-20]. For TCS examination a color-coded phased array ultrasound system equipped with a 1,8-3,6 MHz transducer was used (Sonos 5500, Philips, Netherlands). The examination was performed through the left and right temporal acoustic bone window with a penetration dephth of 16 cm, a dynamic range of 50 dB, and a mechanical index of 1,6. The image brightness was adapted as needed using B-mode gain and lateral gain control. The mesencephalic brainstem was identified by its butterfly shape (figure 1), after image freezing the structure was zoomed two-fold. Within this structure the hyperechogenic signal of the ipsilateral SN was identified. A structure is classified as hyperechogenic if the intensity of the ultrasound signal is abnormally increased compared with a reference structure usually the surrounding white matter. The measurement was carried out after optimisation of the signal at its largest extension. The area of the echogenic SN was surrounded manually with the cursor, the area was calculated automatically. (for examples see figure 2, 3) Median Raphe and ipsilateral red nuclei were identified. In the diencephalic insonation plane the contralateral thalamus was depicted and the width of the third ventricle and the contralateral frontal horn of the lateral ventricle was measured perpendicular at maximal width. The contralateral thalamic, caudate and lentiforme nuclei were classified. The ultrasound examination and measurements were performed according to an international consensus [21]. The whole measurement of SN was repeated by two independent investigators, blinded to the results of each other- one of them an experienced sonographer (SM, ES) and the other a well trained student (PM, KS). The investigators were not blinded to the subjects while scanning but not explicitly informed about the group the subject belonged to.

### Statistics

Descriptive statistics are given as median and lower (25^th ^percentile) and upper (75^th ^percentile) quartile. Correlation analysis was performed by Spearman's rank correlation. For group comparison an unpaired non-parametric data Mann- Whitney-test and for non-parametric data Kruskal-Wallis Test was used. A correction for multiple comparisons was performed where appropriate. For comparison between IPD and NCD group the combined measurements of left and right side were used. The cut-off value was established by Receiver Operating Characteristics (ROC) analysis. Interobserver reliability was calculated as mean ± SD and variation coefficient was estimated.

## Results

In the IPD group transcranial ultrasound could be performed in 183 individuals. 183 of 199 patients (92%) had a sufficient acoustic bone window at least on one side and 15 of these patients only on one side. Their median age was 66 (62;71) years, 24 had a predominant tremor form, 80 akinetic-rigid form and 79 equivalent form. In the NCD group examination was possible unilaterally in all individuals, whereas, bilateral investigation was not possible in 6 of 201 individuals (3%). The median age of NCD individuals was 49 (32;64) years.

In the IPD group median area of the SN was 0.3 cm^2 ^(0.25;0.37) on the left side and 0.29 cm^2 ^(0.25;0.36) on the right side. In the NCD group median area of SN on left side was 0.12 cm^2 ^(0.1;0.15), on the right side 0.12 cm^2 ^(0.09;0.15). The difference of SN sizes between IPD patients and NCD healthy individuals proofed to be highly significant (p < 0.001).

The measurement of SN was found to be adequately reproducible considering the small size of the structure; variation between two observers was small (variation coefficient 0.085). The difference was 0.018 cm^2 ^± 0.015 cm^2^, the range 0.0 cm^2 ^to 0.12 cm^2^. Evaluation concerning group membership applying the cut-off of 0,2 cm^2 ^differed only in 2 NCD and 3 IPD subjects.

The cut-off value for the SN area was established using a Receiver Operating Characteristics (ROC) curve with a sensitivity (true positive rate) of 95%. This corresponded to an area of SN of 0.2 cm^2 ^(AUC 0.989). At this cut-off value specificity in our cohort was 96%.

In the NCD group only 8 of 201 individuals (4%) showed a SN area bilaterally >0,2 cm^2^. An unilateral SN size >0,2 cm^2 ^was found in 13 of 201 individuals (6.5%). (see table 1)

**Table 1:** Clinical and TCS data of NCD group

Age, years	Number of NCD individuals	SN, cm^2^*	Prevalence of SN >0,2 cm^2^, N; in %
20-29	35	0.11 (0.06;0.13)	2; 5.7
30-39	34	0.13 (0.07; 0.15)	1; 2.9
40-49	32	0.11 (0.06; 0.13)	1; 3.1
50-59	36	0.13 (0.07; 0.16)	2; 5.6
60-69	31	0.13 (0.07; 0.16)	0; 0
70-80	33	0.14 (0.05; 0.15)	2; 6.1

*Data are given as median with lower and upper quartile (25th and 75th percentile respectively).

In NCD group SN size increased with age (Spearman Rho = 0.17, p < 0.02), but individuals with SN size >0,2 cm^2 ^did not significantly differ in age from individuals with SN size <0,2 cm^2 ^(Mann-Whitney-U p > 0.5). In NCD group individuals SN size was larger with decreasing hand motor function measured in Pegboard test (Spearman Rho -0.23, p = 0.01), but no significant difference of motor function was found between healthy subjects with normal or increased SN size (Mann-Whitney-U p > 0.1). No correlation was found with Webster's gait test (Spearman Rho 0.13, p > 0.08).

In the IPD patient group only 4.9% (9 of 183 patients) showed a unilateral SN area <= 0,2 cm^2 ^and 3.6% (6 of 168 patients) bilaterally. (see table 2) No significant correlation of SN echogenicity with age (Spearman Rho 0.02, p > 0.7), sex, country of origin, education, contact to toxins, nutrition or concomitant diseases was observed neither in the IPD nor the NCD group. In IPD subjects no differences of SN echogenicity were found between different clinical subtypes (Kruskal-Wallis, Chi^2 ^= 1.9, p > 0.3), age at disease onset (Spearman Rho 0.04, p > 0.5) nor with presentation of fluctuations (Kruskal-Wallis, Chi^2 ^= 0.01, p > 0.9), hyperkinesia (Kruskal-Wallis, Chi^2 ^= 0.05, p > 0.8) or LED (Spearman Rho 0.06, p > 0.4) or start of dopaminergic medication (Spearman Rho 0.01, p > 0.9) (see table 3). SN sizes did not correlate with the clinically more affected body side (Kruskal-Wallis, Chi^2 ^= 0.2, p > 0.3) nor with handedness (Kruskal-Wallis, Chi^2 ^= 1.6, p > 0.2) or Pegboard results (Kruskall-Wallis, Chi^2 ^= 1.3, p > 0.5). No correlation between Hoehn&Yahr stages of disease and SN size was found (Spearman Rho 0.06, p = 0.45). Even though IPD patients with SN>0.2 cm^2 ^had worse results in the Pegboard tests (Mann-Whitney-U p = 0.03), they did not differ in UPDRS (Mann-Whitney-U p = 0.3) or Webster's gait test (Mann-Whitney-U p = 0.37) from test negative (SN <= 0,2 cm^2^) IPD patients.

**Table 2:** Clinical and TCS data of IPD group

Age, year	Number of IPD individuals	SN, cm^2^*	Prevalence of SN <= 0,2 cm^2^, N; in %
40-49	8	0.28 (0.24; 0.39)	0; 0
50-59	27	0.31 (0.28; 0.33)	1; 0
60-69	90	0.30 (0.26; 0.36)	6; 6.7
70-85	58	0.29 (0.25; 0.38)	3; 5.2

*Data are given as median with lower and upper quartile (25th and 75th percentile respectively).

**Table 3:** Clinical data of IPD patients grouped according to SN size in TCS

Data	Whole IPD group* (n = 183)	IPD patients SN <= 0.2 cm^2^* (n = 9)	IPD patients SN>0.2 cm^2^* (n = 174)	p-value (Mann-Whitney-Test)
Age, y	66 (62;71)	69 (67;75)	66 (62;71)	0.18
Age at disease onset, y	61 (54;66)	67 (59;74)	60 (54;66)	0.13
Disease duration, months	78 (48;120)	60 (36;96)	81 (48;120)	0.23
Duration since L-Dopa start, y	5 (3; 8)	3 (1,5;2,2)	5 (3;8)	0.37
L-Dopa equivalent dose, mg	560 (310;679)	519 (306;628)	558 (310;679)	0.83
Hoehn&Yahr score	2.5 (2.5;3)	2.5 (2.5;2.5)	2.5 (2.5;3)	0.88
UPDRS III score	26 (17;34)	27 (20;31)	25 (17;34)	0.28
Pegboard test score	16 (12;20)	13 (13;14)	16 (12;20)	0.03
Webster gait test	13 (10;15)	13 (13;18)	12 (11;15)	0.37

*Data are given as median with lower and upper quartile (25th and 75th percentile respectively). y (years)

Median width of third ventricle in the IPD group was 0.56 cm (0.42;0.73), in the NCD group 0.31 cm (0.22;0.48). The width of the frontal horn of the lateral ventricle was 1.46 cm (1.27;1.69) in IPD on the right side and 1.46 cm on the left side (1.24;1.71). In the NCD group it was 1.15 cm (1.02;1.35) on the right and 1.17 cm (1.02;1.37) on left side. As expected the size of the third and lateral ventricle increased with age in both groups (NCD: Spearman Rho 0.74, p < 0.01; IPD: Spearman Rho 0.42, p < 0.01). Classification of raphe or red nuclei, thalamus, lentiform and caudate nuclei did not differ significantly between NCD and IPD group. (see table 4)

**Table 4:** Qualitative assessment of echogenicity of brainstem raphe, red nucleus, thalamus, caudate and lenticular nuclei in NCD and IPD group

Structure	NCD group (n = 201)		IPD group (n = 183)	
Brainstem raphe	grade 1:n = 23 grade 2:n = 176		grade 1:n = 30 grade 2:n = 130	
	right	left	right	left
Red nucleus	grade 1:n = 44 grade 2:n = 150	grade 1:n = 44 grade 2:n = 154	grade 1:n = 47 grade 2:n = 113	grade 1:n = 49 grade 2:n = 109
Thalamus	grade 1:n = 198 grade 2,3:n = 0	grade 1:n = 198 grade 2,3:n = 0	grade 1:n = 157 grade 2,3:n = 0	grade 1:n = 156 grade 2:n = 1 grade 3:n = 0
Lenticular nucleus	grade 1:n = 194 grade 2:n = 3	grade 1:n = 194 grade 2:n = 4	grade 1:n = 155 grade 2:n = 13 grade 3:n = 1	grade 1:n = 150 grade 2:n = 19 grade 3:n = 3
Caudate nucleus	grade 1:n = 197 grade 2,3:n = 1	grade 1:n = 196 grade 2:n = 2 grade 3:n = 0	grade 1:n = 162 grade 2:n = 3 grade 3:n = 2	grade 1:n = 166 grade 2:n = 4 grade 3:n = 0

Mann-Whitney-U-Test showed no significance between NCD and IPD group for echogenicity of all structures. Structure echogenicity was classified: Raphe: markedly echogenic (2); interrupted or not visible (1). Red nucleus: weakly echogenic (1), markedly echogenic (2), hyperechogenic (3). Thalamus, caudate and lenticular nucleus: iso- or hypoechogenic (1), hyperechogenic (2), markedly hyperechogenic (3).

## Discussion

Transcranial sonography is a valuable method for differentiation of IPD patients from healthy individuals. We could demonstrate a highly characteristic enlargement of SN echogenic signal in patients with IPD. Our results correspond to the findings of other studies. Due to the large group of patients and control subjects studied our results emphasize the importance of this diagnostic tool. The SN echogenic sizes found in our large and homogeneous cohort of individuals without CNS disorder ranging in age from 20 to 79 years correspond to the results in smaller studies reporting a median SN sizes of 0.14 cm^2 ^for healthy subjects aged 50 to 59 years, 0.10 cm^2 ^for subjects 60 to 69 years, and 0.15 cm^2 ^for subjects 70 to 79 years and median SN sizes for patients with nonparkinsonian cerebral disorders of 0.13 cm^2^, although different ultrasound equipment was used [2,8]. Our IPD patients showed a median SN echogenic size of 0.29 cm^2^. This is slightly higher than the reported median SN echogenic size of IPD patients of 0.25 cm^2 ^using Siemens ultrasound devices [2,8].

We set our cut-off value at a sensitivity of 95%. Interestingly, our cut-off value turned out to be equivalent to the cut-off values of other studies, despite differences in the median values using different ultrasound equipment [2,8]. It has to be admitted that in the study of Hagenah et al using the same device SONOS 5500 a lower sensitivity and specificity at a differing cut off of 0,27 cm^2 ^was reported, probably due to the small sample size or due to the different patient sample (Parkin mutation carriers) [12]. Unfortunately formal studies comparing different devices in the same cohort are still lacking. Nevertheless based on our results and considering that in other studies using the same or different ultrasound devices similar scoring criteria concerning SN area were established, the hypothesis that the sonographic method is not substantially influenced by the ultrasound device seems acceptable [11,2,8]. The validity of the scoring criteria, independent of the devices used, is of paramount importance, since this is the basis to advocate TCS as the method of choice in the diagnostic workup of movement disorders.

The dependence of this ultrasound technique on experience and a relative subjectivity of results is an often discussed matter of critique. Regarding the low interobserver variance in our study these arguments might be softend. As documented through our data a good reproducibility can be achieved. Our study design did not allow a blinding towards group membership of individuals included in this study and this may have influenced the results of the study. Given the clinical aspect of IPD patients a sufficient blinding of experienced examiners is almost impossible or would at least require an enormous effort as reported by Prestel et al. [16] We have to admit that our results might be influenced by the age difference of the groups and the lack of a sufficient blinding, so the specificity might be slightly overestimated.

In our study we found a rate of 6.5% of NCD individuals with at least unilaterally increased SN size. A similar rate of about 9% has been reported before [1,2]. Despite the frequency of increased SN size in NCD in our study is higher than the estimated risk of about 0.1% of IPD in the general population it is thought to reflect a vulnerability for developing nigrostriatal disorders [2].

A slight increase of SN size revealed with increasing age and we found worse hand motor function (Pegboard test) correlating with larger SN size. Previously, it has been demonstrated that elderly subjects without prediagnosed extrapyramidal disorder but with increased SN size developed more often signs of motor retardation [3].

Previous studies showed no change of SN sizes in the course of the disease and according to our results no correlation with parameters of disease severity i.e. Hoehn & Yahr stages, UPDRS, Webster-, Pegboard-Test. It is assumed that SN hyperechogenicity reflects an increased amount of iron in the SN, bound to proteins other than ferritin [1,2,9]. It is regarded as a trait marker pointing to a predisposition for the disease not a severitiy marker reflecting proceeding nigral cell loss [21]. Our results show that neither sonographic classification of raphe, thalamus, lenticular or caudate nuclei nor measurement of ventricles could serve as suitable parameters to discriminate healthy individuals from IPD patients. Recent studies showed that these parameters are of paramount importance to distinguish atypical Parkinsonian syndromes from IPD [22].

## Conclusion

Our study shows that TCS is a reliable and highly sensitive tool for differentiation of IPD patients from individuals without CNS disorders. In consideration of other studies our results point to a relative independence of the SN scoring parameters on the ultrasound equipment.

Our study focused on clinicaly diagnosed IPD and did not include atypical Parkinsonian syndromes, this may be seen as a shortcoming. A possible bias influencing our results might be the age difference of the two groups though the investigators were not sufficiently blinded.

## Competing interests

The authors declare that they have no competing interests.

## Authors' contributions

SM, ES, KS, PM carried out the sonography, SM, IR, KS, PM made substantial contribution to the design of the study, contributed to the patient data acquisition and neurologic examinations. SM drafted the manuscript. ES, IR, MK helped in drafting and revision of the manuscript. All authors read and approved the final manuscript.

## Pre-publication history

The pre-publication history for this paper can be accessed here:

http://www.biomedcentral.com/1471-2377/10/9/prepub

## References

[B1] Becker G Seufert J Bogdahn U Reichmann H Reiners K Degeneration of substantia nigra in chronic Parkinson's disease visualized by transcranial color-coded real-time sonography Neurology 1995 45 182 184 7824114 10.1212/wnl.45.1.182

[B2] Berg D Becker G Zeiler B Tucha O Hofmann E Preier M Benz P Jost W Reiners K Lange KW Vulnerability of the nigrostriatal system as detected by transcranial ultrasound Neurology 1999 53 1026 1031 10496262 10.1212/wnl.53.5.1026

[B3] Berg D Siefker C Ruprecht-Dorfler P Becker G Relationship of substantia nigra echogenicity and motor function in elderly subjects Neurology 2001 56 13 17 11148229 10.1212/wnl.56.1.13

[B4] Berg D Roggendorf W Schroder U Echogenicity of the substantia nigra: association with increased iron content and marker for susceptibility to nigrostriatal injury Arch Neurol 2002 56 999 1005 10.1001/archneur.59.6.999 12056937

[B5] Jabs BE Berg D Merschdorf U Bartsch AJ Pfuhlmann B Differences in substantia nigra echogenicity of nosological subtypes within the schizophrenic spectrum. A preliminary transcranial ultrasound study Neuropsychobiology 2001 44 4 183 186 10.1159/000054940 11702018

[B6] Postert T Lack B Kuhn W Basal ganglia alterations and brain atrophy in Huntington's disease depicted by transcranial real time sonography J Neurol Neurosurg Psychiatry 1999 67 457 462 10.1136/jnnp.67.4.457 10486391 PMC1736595

[B7] Ruprecht-Dörfler P Berg D Tucha O Benz P Meier-Meitinger M Alders GL Lange KW Becker G Echogenicity of the substantia nigra in relatives of patients with sporadic Parkinson's disease Neuroimage 2003 18 2 416 422 10.1016/S1053-8119(02)00035-6 12595195

[B8] Walter U Wittstock M Benecke R Dressler D Substantia nigra echogenicity is normal in non-extrapyramidal cerebral disorders but increased in Parkinson's disease J Neural Transm 2002 109 191 196 10.1007/s007020200015 12075859

[B9] Walter U Niehaus L Probst T Benecke R Meyer BU Dressler D Brain parenchyma sonography discriminates Parkinson's disease and atypical parkinsonian syndromes Neurology 2003 60 1 74 77 12525721 10.1212/wnl.60.1.74

[B10] Okawa M Miwa H Kajimoto Y Hama K Morita S Nakanishi I Kondo T Transcranial sonography of substantia nigra in Japanese patients with Parkinson's disease or atypical Parkinsonism: clinical potential and limitations Intern Med 2007 46 18 1527 31 10.2169/internalmedicine.46.0271 17878638

[B11] Vlaar AM de Nijs T van Kroonenburgh MJ Mess WH Winogrodzka A Tromp SC Weber WE The predictive value of transcranial duplex sonography for the clinical diagnosis in undiagnosed parkinsonian syndromes: comparison with SPECT scans BMC Neurol 2008 8 42 10.1186/1471-2377-8-42 18992168 PMC2628347

[B12] Hagenah J M König I R Becker B Hilker R Kasten M Hedrich K Pramstaller P P Klein C Seidel G Substantia nigra hyperechogenicity correlates with clinical status and number of Parkin mutated alleles J Neurol 2007 254 1407 1413 10.1007/s00415-007-0567-y 17934880

[B13] Hagenah J M Becker B Bruggemann N Djarmati A Lohmann K Sprenger A Klein C Seidel G Transcranial sonography findings in a large family with homozygous and heterozygous PINK1 mutations J Neurol Neurosurg Psychiatry 2008 79 1071 1074 10.1136/jnnp.2007.142174 18469032

[B14] Hughes AJ Daniel SE Kilford L Lees AJ Accuracy of clinical diagnosis of idiopathic Parkinson's disease: a clinico-pathological study of 100 cases J Neurol Neurosurg Psychiatry 1992 55 3 181 184 10.1136/jnnp.55.3.181 1564476 PMC1014720

[B15] Fahn S Elton RL Members of the UPDRS Development Committee Fahn S, Marsden CD, Calne DB, Goldstein M The Unified Parkinson's Disease Rating Scale Recent developments in Parkinson's disease 1987 2 Florham Park, NJ: Macmillan Health Care Information 153 163 293-304.

[B16] Webster DD Critical analysis of the disability in Parkinson's disease Mod Treat 1968 5 257 282 5655944

[B17] Vingerhoets FJ Schulzer M Calne DB Snow BJ Which clinical sign of Parkinson's disease best refelcts the nigrostriatal lesion? Ann Neurol 1997 41 1 58 64 10.1002/ana.410410111 9005866

[B18] Maschke M Gomez CM Tuite PJ Dysfunction of the basal ganglia, but not the cerebellum, impairs kinaesthesia Brain 2003 126 2312 22 10.1093/brain/awg230 12821507

[B19] Hobson DE Lang AE Martin WR Excessive daytime sleepiness ans sudden onset sleep in Parkinson disease: a survey by the Canadian Movement Disorders Group JAMA 2002 287 455 63 10.1001/jama.287.4.455 11798367

[B20] Parkin SG Gregory RP Scott R Unilateral and bilateral palidotomy for idiopathic Parkinson's disease: a case series of 115 paients Mov Disord 2002 17 682 92 10.1002/mds.10186 12210857

[B21] Walter U Behnke S Eyding J Niehaus L Postert T Seidel G Berg D Transcranial brain parenchyma sonography in movement disorders: state of the art Ultrasound Med Biol 2007 33 1 15 25 10.1016/j.ultrasmedbio.2006.07.021 17189043

[B22] Prestel J Schweitzer KJ Hofer A Gasser T Berg D Predictive value of transcranial sonography in the diagnosis of Parkinson's disease Mov Dis 2006 21 10 1763 1765 10.1002/mds.21054 16874758

